# Surveillance for severe acute respiratory infections in Southern Arizona, 2010–2014

**DOI:** 10.1111/irv.12360

**Published:** 2016-01-29

**Authors:** Zimy Wansaula, Sonja J. Olsen, Mariana G. Casal, Catherine Golenko, Laura M. Erhart, Peter Kammerer, Natalie Whitfield, Orion Z. McCotter

**Affiliations:** ^1^ Arizona Department of Health ServicesOffice of Border HealthTucsonAZUSA; ^2^ Influenza DivisionCenters for Disease Control and PreventionAtlantaGAUSA; ^3^ Arizona Department of Health ServicesOffice of Infectious Disease ServicesPhoenixAZUSA; ^4^ Naval Health Research CenterSan DiegoCAUSA; ^5^ Clinical and Molecular MicrobiologyUniversity of ArizonaTucsonAZUSA; ^6^ Mycotic Diseases BranchCenters for Disease Control and PreventionAtlantaGAUSA

**Keywords:** Arizona, influenza, respiratory tract diseases, surveillance

## Abstract

**Background:**

The Binational Border Infectious Disease Surveillance program began surveillance for severe acute respiratory infections (SARI) on the US–Mexico border in 2009. Here, we describe patients in Southern Arizona.

**Methods:**

Patients admitted to five acute care hospitals that met the SARI case definition (temperature ≥37·8°C or reported fever or chills with history of cough, sore throat, or shortness of breath in a hospitalized person) were enrolled. Staff completed a standard form and collected a nasopharyngeal swab which was tested for selected respiratory viruses by reverse transcription polymerase chain reaction.

**Results:**

From October 2010–September 2014, we enrolled 332 SARI patients. Fifty‐two percent were male and 48% were white non‐Hispanic. The median age was 63 years (47% ≥65 years and 5·2% <5 years). During hospitalization, 51 of 230 (22%) patients required intubation, 120 of 297 (40%) were admitted to intensive care unit, and 28 of 278 (10%) died. Influenza vaccination was 56%. Of 309 cases tested, 49 (16%) were positive for influenza viruses, 25 (8·1%) for human metapneumovirus, 20 (6·5%) for parainfluenza viruses, 16 (5·2%) for coronavirus, 11 (3·6%) for respiratory syncytial virus, 10 (3·2%) for rhinovirus, 4 (1·3%) for rhinovirus/enterovirus, 3 (1·0%) for enteroviruses, and 3 (1·0%) for adenovirus. Among the 49 influenza‐positive specimens, 76% were influenza A (19 H3N2, 17 H1N1pdm09, and 1 not subtyped), and 24% were influenza B.

**Conclusion:**

Influenza viruses were a frequent cause of SARI in hospitalized patients in Southern Arizona. Monitoring respiratory illness in border populations will help better understand the etiologies. Improving influenza vaccination coverage may help prevent some SARI cases.

## Introduction

The 2009 influenza pandemic highlighted the need for more global data on severe influenza disease, and the World Health Organization recommended Member States conduct surveillance for hospitalized severe acute respiratory infection (SARI) in addition to surveillance for influenza‐like illness (ILI) in outpatients.^1^ As a result, SARI surveillance is now conducted in many countries around the world; however, it is only conducted in limited settings in the United States. The pandemic also highlighted the importance of having surveillance on the US–Mexico border, as the first cases of influenza A(H1N1)pdm09 virus infection were detected in southern California.^2^, ^3^

The Arizona Department of Health Services (ADHS) has conducted statewide influenza surveillance since 1997. Surveillance indicators include monitoring ILI among ambulatory patients, tracking laboratory‐confirmed cases, monitoring ILI in schools, and testing and subtyping influenza viruses in specimens submitted to the Arizona State Public Health Laboratory. Laboratory‐confirmed cases of respiratory syncytial virus (RSV) infections are also monitored. However, epidemiological data on influenza hospitalizations has been limited, and there is no established statewide surveillance for respiratory viral infections beyond influenza and RSV. In 2010, Arizona began conducting SARI surveillance as part of Centers for Disease Control and Prevention (CDC) Binational Border Infectious Disease Surveillance (BIDS) program with the aim of describing the patterns of disease in a highly fluid border region. Here, we report SARI surveillance data in Arizona from 2010 to 2014.

## Methods

The ADHS’ Human Subjects Review Board determined this surveillance was part of public health practice.

### Study sites

In 2010, the ADHS, BIDS program initiated SARI surveillance at selected hospitals in the Arizona region that borders Mexico. We defined Southern Arizona as four counties (Cochise, Pima, Santa Cruz, and Yuma). There were 11 acute care hospitals within this area; surveillance was initiated at five hospitals: St. Mary's Hospital (adults), St. Joseph's Hospital (adults), Tucson Heart Hospital (adults), Northwest Medical Center (adults), and Sells Indian Health Services Clinic (all ages). These sites were selected because they routinely receive acutely ill patients and transferred patients from smaller hospitals in the border area. Two hospitals (Tucson Heart and Northwest Medical) had fewer than five participants in the first season and were dropped in future seasons. Of the six hospitals not included, four declined to participate, one was not approached as it serves a retirement community, and one was excluded because it did not have an emergency room.

### Case ascertainment

A clinical team at each hospital was trained on the case definition and surveillance procedures; patients were asked for verbal consent to participate. Any patients who presented at the emergency ward and were identified by the clinical hospital team as meeting the SARI case definition and consented were enrolled. A case of SARI was defined as temperature ≥37·8°C or subjective fever or chills, in addition to cough, sore throat, or shortness of breath in a patient requiring hospital admission. Additionally, children <5 years old were included if they had a clinical suspicion of pneumonia requiring hospital admission. All patients meeting the case definition were enrolled; surveillance was conducted all 7 days of the week.

Patients meeting the SARI case definition were given a unique identification number, and a one‐page standardized questionnaire was completed that included demographic information, clinical signs and symptoms, comorbidities, reported influenza vaccine status, rapid influenza results, and recent travel history. A trained nurse collected a nasopharyngeal swab from each participant. For two sites, an electronic medical record system was searched to obtain missing data or patient outcome.

### Laboratory methods

Nasopharyngeal swabs were placed into 3 ml of universal viral transport media. The specimens were refrigerated at the hospital at 4°C and delivered daily by courier service to the reference laboratory at the University of Arizona, Infectious Disease Research Core. Specimens were tested for viral pathogen targets by a multiplex polymerase chain reaction (PCR) using the ResPlex II assay (v. 2.0) (Qiagen, Hilden, Germany) from 2010 to 2012 or the GenMark Respiratory Viral Panel assay from 2013 to 2014 (GenMark Diagnostics Inc., Carlsbad, CA, USA).^4^, ^5^ Assay viral targets included adenoviruses, bocaviruses (only 2010–2012), coronaviruses (229E, OC43, NL63, HKU1) (only 2010–2012), enteroviruses (only 2010–2012), human metapneumovirus (HMPV), influenza viruses A and B, parainfluenza viruses (PIV) (PIV1‐4 2010‐2012; PIV1‐3 2013‐2014), RSV A and B, and rhinoviruses. The ResPlex II assay identified a broad range of enteroviruses; there was some cross‐reactivity between the targets for enteroviruses and rhinoviruses, so these are reported as rhinovirus/enterovirus.

Laboratory results were faxed back to the sites so that physicians could use the surveillance results to understand the epidemiology of circulating respiratory infections. The remaining portion of the specimens was frozen at −80°C and sent on dry ice to the Naval Health Research Center (NHRC) in San Diego for testing by real‐time polymerase chain reaction (rRT‐PCR) for influenza virus type and subtype, RSV, adenoviruses, and rhinoviruses (starting in 2011) using singleplex PCR on the ABI7500 platform (Applied Biosystems, Foster City, CA, USA).^6^, ^7^ We report influenza, RSV, adenovirus, and rhinovirus results from the NHRC; all other results are from the University of Arizona laboratory. Specimens positive for an enterovirus and a rhinovirus from different laboratories were also reported as rhinovirus/enterovirus.

### Data analysis

Case report forms and laboratory results were entered into Epi Info 7.1 (Centers for Disease Control and Prevention) and managed by BIDS staff. We analyzed data from 4 years (week 40, 2010–week 39, 2014). An influenza season began in week 40 and ended in week 39 of the consecutive year. Data analyses were conducted using SAS statistical software version 9.3 (SAS Institute Inc, Cary, NC, USA). Differences in the proportion of a characteristic between groups of patients were compared with the chi‐square test; a Fisher exact test was used to account for small sample sizes of some variables. All tests were two‐sided, and differences with *P* values <0·05 were considered significant.

## Results

### Patient demographics

From October 2010 to September 2014, 406 patients were reported to BIDS by the surveillance hospital sites. Of the 406 patients, 74 (18%) did not meet the SARI case definition and were excluded from further analyses. Of the 332 cases analyzed, 174 (52%) were in male patients (Table 1). The median age was 63 years (range: 0–97 years). The age distribution was skewed toward older groups, with 156 (47%) aged ≥65 years, 80 (24%) aged 50–64 years, 67 (20%) aged 25–49 years, 10 (3%) aged 5–24 years, and 17 (5·2%) aged <5 years. Most cases were in persons who were White non‐Hispanic (*n* = 160, 48%) or Hispanic (*n* = 118, 36%). Fourteen (4·4%) reported to have travelled within 10 days of symptom onset, 10 (3·2%) to Mexico and four (1·3%) to other countries.

**Table 1:** Demographic and clinical characteristics of patients hospitalized with severe acute respiratory infection (SARI) cases in Arizona, 2010–2014 (*n* = 332)

Characteristics	Enrolled SARI cases (*n* = 332) *N* (%)	Influenza viruses (*n* = 49) *N* (%)	Human metapneumovirus (*n* = 25) *N* (%)	Parainfluenza viruses (*n* = 20) *N* (%)	Coronavirus (*n* = 16)^a^ *N* (%)	Respiratory syncytial virus (*n* = 11) *N* (%)
Demographics						
Male	174 (52)	19 (39)	14 (56)	13 (65)	7 (44)	3 (27)
Median age in years (range)	63 (0–97)	62 (1–97)	65 (0–89)	64 (0–86)	80 (29–88)	3 (0–78)
Age 0–4 years	17 (5·2)	2 (4·1)	2 (8·0)	3 (15)	0	6 (55)
5–24	10 (3·0)	1 (2·0)	3 (12)	1 (5·0)	0	0
25–49	67 (20)	12 (25)	2 (8·0)	1 (5·0)	3 (19)	1 (9·1)
50–64	80 (24)	13 (27)	5 (20)	6 (30)	1 (6·3)	1 (9·1)
≥65	156 (47)	21 (43)	13 (52)	9 (45)	12 (75)	3 (27)
Race/Ethnicity						
White non‐Hispanic	160 (48)	23 (47)	11 (44)	8 (40)	10 (63)	0
Hispanic/Latino	118 (36)	20 (41)	7 (28)	8 (40)	5 (31)	5 (45)
American Indian/Alaska Native	41 (12)	5 (10)	5 (20)	4 (12)	1 (6·3)	6 (55)
Black/African American	8 (2·4)	0	2 (8·0)	0	0	0
Asian	4 (1·2)	1 (2)	0	0	0	0
Signs/Symptoms at admission						
Cough	300 (90)	48 (98)	25 (100)	19 (95)	12 (75)	10 (91)
Shortness of breath	254 (77)	37 (76)	19 (76)	15 (75)	14 (88)	8 (73)
Fever (T ≥ 37·8°C)	240 (72)	37 (76)	19 (76)	18 (90)	11 (69)	10 (91)
Clinical suspicion of pneumonia	232 (71)	30 (61)	20 (80)	17 (85)	11 (69)	3 (28)
Sputum production	174 (52)	30 (61)	16 (64)	9 (45)	6 (38)	5 (45)
Chills	164 (49)	26 (53)	11 (44)	10 (50)	5 (32)	4 (36)
Feverish	121 (37)	22 (45)	13 (52)	2 (10)	9 (53)	0
Body ache	109 (33)	16 (33)	9 (33)	5 (25)	4 (25)	3 (27)
Wheezing	94 (28)	13 (27)	10 (40)	6 (30)	4 (25)	4 (36)
Nasal congestion	92 (28)	16 (33)	9 (36)	6 (30)	4 (25)	4 (36)
Nausea or vomiting	88 (27)	16 (33)	5 (21)	3 (15)	4 (25)	3 (27)
Headache	86 (26)	16 (33)	3 (12)	4 (20)	1 (6·3)	2 (18)
Sore throat	76 (23)	11 (23)	5 (20)	6 (30)	2 (13)	4 (36)
Diarrhea	47 (14)	10 (20)	0	1 (5)	0	3 (27)
Comorbidities						
Hypertension	136 (41)	24 (49)	10 (40)	9 (20)	11 (69)	1 (9·1)
Metabolic disorder	109 (33)	22 (45)	7 (28)	7 (35)	4 (25)	2 (18)
Chronic lung disease	99 (30)	16 (33)	3 (12)	5 (25)	8 (50)	2 (18)
Cardiac disease	80 (24)	14 (29)	7 (28)	9 (45)	4 (25)	0
Current smoker	41/208 (20)	5 (13)	3/18 (17)	2/10 (20)	1 (11)	0
Immunosuppression	35 (11)	2 (4)	3 (13)	0	1 (6·3)	0
Morbid obesity	37 (11)	6 (12)	3 (12)	2 (10)	2 (13)	1 (9·1)
Neuromuscular disease	18 (5·4)	1 (2)	3 (12)	1 (5)	2 (12)	0
Outcomes						
Admission to intensive care unit	120/297 (40)	14 (33)	4/22 (18)	6 (40)	6 (38)	2/6 (33)
Intubation	51/230 (22)	11 (29)	1/18 (5·6)	0	4 (25)	NA
Death	28/278 (10)	3 (6·4)	2/23 (8·7)	2/16 (13)	3 (19)	0/5

NA, not available.

a One patient was co‐infected with coronavirus OC43 and coronavirus HKU1.

Among those with an influenza vaccination history, 56% (*n* = 138/245) self‐reported vaccination within the 12 months prior to hospital admission. The lowest vaccination rate was observed among Native Americans (41%, *n* = 11/27) and patients aged 25–49 years (36%, *n* = 16/45). Patients aged ≥65 years reported receiving influenza vaccination (73%) more often than patients younger than 65 years (42%) (*P* < 0·0001). If we assume that patients who reported ‘unknown’ for influenza vaccination status did not receive a vaccination, overall coverage dropped to 42% (138/332) and from 64% to 49% among White non‐Hispanic, 50% to 39% among Hispanic, 50% to 33% among Asian, and 41% to 29% among Native American.

### Clinical characteristics

The majority of SARI patients presented at admission with cough (90%), fever (72%), shortness of breath (77%), and clinical suspicion of pneumonia (71%) (Table 1). Of 237 patients with documentation of a chest radiograph, 132 (56%) had radiographic evidence of pneumonia. Seventy‐six percent of patients reported having at least one underlying medical condition. The most common comorbidities reported were hypertension (41%), metabolic disorders (33%), and chronic lung disease (30%). Patients with comorbidities (*n* = 251, 76%) were older compared to patients with no comorbidities (median age: 68 versus 38) (Table S1). Additionally, they were statistically significantly less likely to present with nasal congestion, body ache, wheezing, and sore throat. The median number of days between symptom onset and hospital admission was four days (interquartile range: 2–7 days). The average length of hospital stay was seven days (range: 1–57 days). Thirty‐six patients (11%) received oseltamivir. Among SARI patients, 40% were admitted to an intensive care unit (ICU), 22% were intubated, and 10% died while hospitalized (Table 1). Of the 51 patients intubated, 39 (77%) had at least one medical comorbidity; 16 of 46 (35%) died. Overall mortality in SARI patients was 10% and peaked at 15% in 2010–2011. During 2010–2011, patients that died were all aged ≥65 years and had comorbidities. Three of these patients tested positive for viral pathogens (two coronaviruses and one parainfluenza). In all seasons combined, of SARI patients that died, 93% were aged ≥50 years, 93% had a comorbidity, and 96% were admitted to the ICU. Of the 28 patients that died, 11 (39%) tested positive for a viral pathogen; three were positive for a coronavirus, three for an influenza virus, two for a HMPV, two for a parainfluenzavirus, and one for a rhinovirus.

### Laboratory testing results

Of the 332 SARI cases, 309 (93%) patients had adequate specimens obtained for respiratory testing, and 134 (43%) tested positive for 142 viral respiratory pathogens: 49 (16%) were positive for influenza viruses, 25 (8·1%) for HMPV, 20 (6·5%) for PIV, 16 (5·2%) for coronaviruses, 11 (3·6%) for RSV, 10 (3·2%) for rhinoviruses, four (1·3%) for rhinovirus/enterovirus, three (1·0%) for enteroviruses, three (1·0%) for adenoviruses, and zero for bocaviruses (Table 2). Specimens were taken an average of 8 days after illness onset (range: 1–90 days). Patients whose specimens were collected within 7 days of the onset of symptoms were more likely to have a viral pathogen detected than those whose specimens were collected later (68% versus 32%, *P* = 0·038). There were no differences in the clinical presentation of patients who tested positive for viral pathogens and those who tested negative except that the latter were more likely to be admitted to an ICU (Table S2). Of the 233 patients with information on influenza vaccination and laboratory testing, 132 (57%) were vaccinated. Of these 132 patients, 76 (58%) tested negative for a virus.

**Table 2:** Respiratory viruses identified among patients hospitalized with severe acute respiratory infection in Arizona, 2010–2014 (*n* = 309)

Respiratory virus	*N* (%)
Influenza viruses	49 (16)
A (not subtyped)	1
A (H1N1)pdm09	17
A (H3N2)	19
B	12
Human metapneumovirus	25 (8·1)
Rhinoviruses/Enteroviruses	17 (5·5)
Rhinoviruses	10
Enteroviruses	3
Rhinovirus/enterovirus	4
Parainfluenza viruses (PIV)	20 (6·5)
PIV1	5
PIV2	2
PIV3	10
PIV4	3
Coronaviruses	17 (5·5)
OC43	9
HKU1	4
NL63	3
229E	1
Respiratory syncytial virus	11 (3·6)
Adenoviruses	3 (1·0)
Bocaviruses	0
Total viruses detected	142
Cases with one virus	127
Cases with two viruses^a^	6
Cases with three viruses^b^	1
Any virus detected	134 (43%)
No virus detected	175 (57%)

a Rhinovirus/coronavirus (*n* = 2), coronavirus HKU1/coronavirus OC43, parainfluenza/adenovirus, parainfluenza/RSV, RSV/enterovirus.

b Influenza B/coronavirus HKU1/human metapneumovirus.

**Figure 1:**
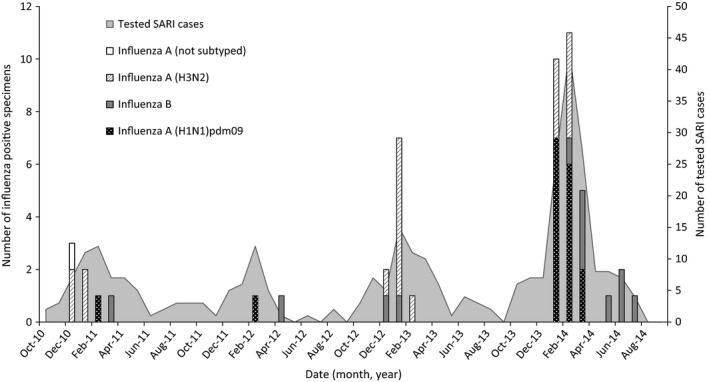
Number and percent of patients hospitalized with severe acute respiratory infections that were positive for influenza viruses by type/subtype and month, Arizona, 2010–2014.

Among the 49 influenza‐positive specimens, 76% were influenza A and 24% influenza B. While influenza A was more commonly detected in the beginning of the season (December to March), influenza B was detected throughout the season and was the only influenza virus identified between April and July (Figure 1). The percentage of SARI samples that was positive for an influenza virus was 11% in 2010–2011, 5·6% in 2011–2012, 15% in 2012–2013, and 20% in 2013–2014. In the 2010–2011 season, of the seven influenza viruses, four (57%) were influenza A (H3N2), one (14%) was influenza A(H1N1)pdm09, one was influenza A non‐subtyped (14%), and one was influenza B (14%); in the 2011–2012 season, of the two influenza viruses, one was influenza A(H1N1)pdm09 (50%) and one was influenza B (50%); in the 2012–2013 season, of the 10 influenza viruses, eight (80%) were influenza A (H3N2) and two were influenza B (20%), and in the 2013–2014 season, of the 30 influenza viruses, 15 (50%) were influenza A(H1N1)pdm09, eight (27%) were influenza B, and seven (23%) were influenza A (H3N2). Thirteen (27%) of the SARI patients with an influenza virus received oseltamivir, and 10 of the 13 (77%) received it on the day of admission; three persons received it within 48 hours of illness onset.

**Figure 2:**
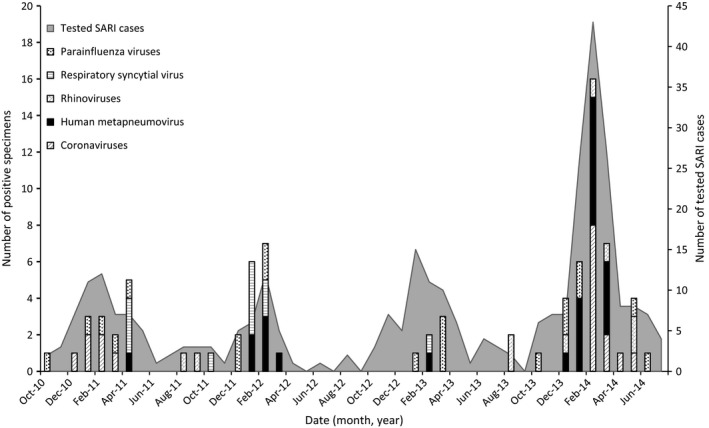
Number of non‐influenza respiratory viruses in patients hospitalized with severe acute respiratory infection, Arizona, 2010–2014.

The monthly distribution of non‐influenza respiratory viruses is shown in Figure 2. Their monthly distribution was similar to that of influenza viruses. Overall, 89% (*n* = 86) of respiratory viruses were identified between October and May. February had the highest number of viruses detected, especially for coronavirus (10/18, 56%) and HMPV (11/24, 46%). Over the four seasons, HMPV was the second most frequently detected virus circulating and the highest annual proportion of all HMPVs (71%) was seen in the 2013–2014 season. All four parainfluenza virus types were detected, but type 3 (50%) dominated.

Compared to patients who tested positive for other viral pathogens, patients who tested positive for an influenza virus were significantly more likely to be intubated (29% versus 11%, *P* = 0·032), have a headache (33% versus 18%, *P* = 0·047), or have diabetes (47% versus 22%, *P* = 0·03) (Table 3). Compared to patients with no respiratory virus identified, patients with an influenza virus were significantly more likely to be female (61% versus 45%, *P* = 0·047), have diabetes (47% versus 21%, *P* = 0·007), or have renal disease (30% versus 6%, *P* = 0·001). Patients with an influenza virus were significantly more likely to present within seven days of symptom onset compared to patients with no respiratory virus identified (94% versus 73%, *P* = 0·003).

**Table 3:** Comparison of patients hospitalized with severe acute respiratory infection with an influenza virus to those with a non‐influenza respiratory viruses or to those with no respiratory virus identified, Arizona, 2010–2014

Characteristics	Influenza virus positive (*n* = 49) *N* (%)	Other respiratory virus positive (*n* = 85) *N* (%)	*P* value^a^	No respiratory virus identified (*n* = 175) *N* (%)	*P* value^b^
Female	30 (61)	40 (47)	0·11	79 (45)	0·047
Cough	48 (98)	77 (91)	0·15	156 (89)	0·15
Fever	37 (76)	67 (79)	0·66	121 (69)	0·39
Shortness of breath	37 (76)	66 (77)	0·78	135 (77)	0·81
Clinical suspicion of pneumonia	30 (63)	59 (69)	0·40	128/173 (74)	0·12
Chills	26 (53)	35 (41)	0·18	92 (53)	0·95
Sore throat	11 (22)	24 (28)	0·46	34 (19)	0·64
Headache	16 (33)	15 (18)	0·047	48 (27)	0·47
Wheezing	13 (27)	30 (35)	0·29	45 (26)	0·9
Nasal congestion	16 (33)	25 (29)	0·69	46 (26)	0·39
Diarrhea	10 (20)	8 (9·4)	0·07	25 (14)	0·29
Feverish	22 (45)	26 (31)	0·09	65 (37)	0·32
Nausea or vomiting	16 (33)	19 (22)	0·19	48 (27)	0·47
Hypertension	24 (49)	30 (35)	0·56	73 (42)	0·36
Diabetes	14/30 (47)	8/37 (22)	0·03	16/77 (21)	0·007
Chronic lung disease	16 (33)	23 (27)	0·49	55 (31)	0·87
Cardiac disease	14 (29)	22 (26)	0·7	39 (22)	0·36
Renal diseases	9/30 (30)	5/37 (55)	0·09	4/77(6)	0·001
Morbid obesity	6 (12)	6 (7·1)	0·3	22 (13)	0·95
Immunosuppression	2 (4·1)	6 (7·1)	0·7	25 (14)	0·08
Admission to intensive care unit	14/43 (33)	22/71 (31)	0·86	76/162 (47)	0·66
Mechanical intubation	11/38 (29)	5/47 (11)	0·03	33/130 (25)	0·7
Death	3/47 (6·4)	8/67 (12)	1·00	16/148 (11)	0·52
Symptom onset <7 days	44/47 (94)	70/83 (84)	0·17	122/167 (73)	0·003

a *P* value for test for difference between influenza and other respiratory virus.

b *P* value for test for difference between influenza and no virus identified.

Compared to SARI patients of other ages, children <5 years of age were significantly more likely to have a respiratory virus detected (82% versus 41%, *P* < 0·0016). Among children <5 years positive for a respiratory virus, RSV was the most commonly identified virus (35%, *n* = 6/17). Additionally, 50% of mixed viral infections were observed among this age group (*n* = 3/6): enteroviruses/RSV (*n* = 1), RSV/PIV3 (*n* = 1), and adenovirus/PIV 3 (*n* = 1).

Among the 51 patients intubated, 16 (31%) tested positive for a viral respiratory pathogen. Of these 16 intubated cases, influenza virus was the most common (69%), followed by rhinovirus (13%), coronavirus (13%), and HMPV (6%). Eleven (39%) patients that died had a viral pathogen identified [influenza (*n* = 3), coronavirus (*n* = 3), HMPV (*n* = 2), parainfluenza virus (*n* = 2), and rhinovirus (*n* = 1)]. None of the three patients with an influenza virus reported being vaccinated for influenza.

## Discussion

This surveillance provides an epidemiological picture of severe respiratory illness and influenza activity in Southern Arizona, highlighting the importance of influenza viruses. As seen elsewhere, influenza viruses were a major contributor (16%) to viral respiratory infections associated with SARI.^8^, ^9^, ^10^ Additionally, this surveillance identified and highlighted the importance of other, often neglected, viral respiratory pathogens including PIV, coronaviruses, and HMPV. Although children <5 years of age accounted for only a small proportion of our population (5·5%), they were more likely to test positive for viral infections than other age groups, and most of their infections were due to RSV, which is the most common cause of acute respiratory infections in children globally.^11^

Few SARI surveillance systems in other countries tested for viral pathogens other than influenza, and those that do often focused on children <5 years old.^12^, ^13^, ^14^ The overall detection rate of viral pathogens among hospitalized SARI cases aged <5 years in our surveillance (82%) was lower than in China (94% in <72 months)^14^ and higher than in Kenya (71%) or in Bangladesh (52%).^11^, ^12^ The proportion of SARI cases that were positive for RSV in children <5 years in our surveillance (31%) was similar to surveillance data in Bangladesh (37%) and slightly higher than in Kenya (21%). Some differences in case definitions or surveillance design (population‐based or hospital‐based) may explain differences observed in the results. The proportion of SARI cases <5 years that were positive for an influenza virus (11%) was also similar to that found in Thailand (13%) and 15 countries across Africa (10%).^8^, ^15^ Besides RSV and influenza viruses, our results also showed that parainfluenza, HMPV, coxsackievirus, and echovirus are important etiologies of SARI in young children, a finding consistent with other studies.^12^, ^13^, ^14^ The finding that 57% of SARI cases had no pathogen detected suggests that other causes (e.g., bacteria) may play an important role in SARI; alternatively, poor or late specimen collection may have contributed to a lower yield in the viruses being detected.

In Southern Arizona, clinical presentation was similar across etiologies of SARI, and only headache was more frequently reported in persons with an influenza virus compared to persons with another viral pathogen. The similar clinical presentation across etiologies highlights the challenges to clinicians. Despite the frequent detection of influenza viruses, few patients received antivirals and even fewer within the recommended time frame highlighting the need to increase empiric antiviral treatment in severe illness.^16^ As expected, persons who had a specimen taken earlier in their illness course were more likely to yield a positive influenza result, highlighting the decrease in shedding over time.^17^ In our surveillance, only three patients had influenza virus detected seven days after the onset of symptoms.

Overall mortality in hospitalized SARI patients was high (10%), and although the numbers are small, 18% of persons with a coronavirus, 13% with a parainfluenza virus, and six percent with an influenza virus died. The high mortality rate may reflect both the older age of our surveillance population (median age 63 years) and the increasing prevalence of underlying disease with age. More than 89% of all SARI patients aged ≥50 years old had preexisting medical conditions and 93% of those who died while hospitalized were ≥50 years old. As has been shown by others, comorbidities such as chronic lung disease, diabetes, and hypertension likely contributed to the poor survival of patients with influenza in our population.^18^ Although we do not know the reason for the slightly higher SARI mortality in the 2010–2011 season, it is consistent with an influenza season that had higher rates of hospitalization in persons ≥65 years. Influenza vaccine coverage in the patients hospitalized with SARI was similar to statewide coverage estimates in Arizona (annual range: 38%–42%).^19^ However, more work can be done to improve vaccination rates overall and in vulnerable populations.

The seasonality of influenza in this SARI surveillance system mimicked national patterns in the United States, with activity peaking in the winter months (December–February), as opposed to the slightly more diffuse patterns observed throughout Mexico.^20^ Influenza B viruses more commonly circulated after influenza A viruses in these four seasons. All influenza virus subtypes detected were known to be in circulation in Arizona, the United States and Sonora, Mexico (Personal Communication Sonora Health Ministry).^21^

This surveillance system has several limitations. First, our selection of large, referral hospitals likely biased our sample to more severe cases presenting later in the course of illness. Further, two of the six hospitals in Southern Arizona that were not included may be more likely to see mobile populations as they are physically closer to border. Although the surveillance was intended to be comprehensive within the selected hospitals, it might not have captured all SARI cases that presented at the hospital. Some cases may have not been enrolled due to doctors’ omission or patients’ admission without passing through the emergency ward. Additionally, few children <5 years were enrolled as our sites are not major providers of pediatric care in Southern Arizona. This population might not be representative of the population of Southern Arizona, and its age distribution may underestimate the burden of SARI, especially among children. Also, because we collected information on mortality at the hospital, deaths that occurred in patients discharged to hospices or nursing homes for terminal care were not included. However, we are in the process of analyzing death certificate data to identify deaths associated with the hospitalization in order to get a better understanding of the predictors of mortality and the mortality burden associated with SARI in Southern Arizona. Self‐reported characteristics such as vaccination, travel information, and symptoms may be subject to poor recall, but we reduced recall bias by verifying information through medical records review for the two major sites with accessible medical records. Finally, a few of the viral targets were only tested for two of the four years of the surveillance, so the proportion positive may be underestimated.

The U.S–Mexico border is porous; an estimated 22·9 million people legally crossed the northbound Arizona border in 2014.^22^ Mobile populations present challenges to the prevention and control of infectious respiratory diseases, as evidenced by the recent outbreak of pneumococcal disease and influenza in unaccompanied children coming to the United States.^23^ Surveillance in border regions is essential to better monitor changing disease patterns and identify focus areas for prevention. The SARI surveillance system described here will continue to yield important information on the etiologies and seasonality of severe respiratory illness in Arizona. This information is important to clinicians who often treat respiratory illness symptomatically. Further, the data suggesting low influenza vaccination coverage suggests that efforts to improve messages on annual influenza vaccination may be needed.

## Competing interests

The authors have no competing interests.

## Disclosures

The findings and conclusions in this report are those of the authors and do not necessarily represent the official position of the U.S. Centers for Disease Control and Prevention or Arizona Department of Health Services.

## Supporting information

Table S1. Comparison of patients hospitalized with severe acute respiratory infection with comorbidity to those with no comorbidity, Arizona, 2010–2014.
Table S2. Comparison of patients hospitalized with severe acute respiratory infection with a viral pathogen to those with no viral pathogen identified, Arizona, 2010–2014.
