# Supplementary material for: Surveillance for severe acute respiratory infections in Southern Arizona, 2010–2014
Source: Influenza Other Respir Viruses. 2016 Jan 29;10(3):161–9. doi: 10.1111/irv.12360 (PMC4814863; doi:10.1111/irv.12360)
Supplement: Supplementary file 1 — Table S1. Comparison of patients hospitalized with severe acute respiratory infection with comorbidity to those with no comorbidity, Arizona, 2010–2014. Table S2. Comparison of patients hospitalized with severe acute respiratory infection with a viral pathogen to those with no viral pathogen identified, Arizona, 2010–2014. [file IRV-10-161-s001.docx]

Supplemental Table 1. Comparison of patients hospitalized with severe acute respiratory infection with comorbidity to those with no comorbidity, Arizona, 2010-2014

| Characteristics | SARI cases with comorbidities  (n=251) n (%) | SARI cases with no comorbidities  (n=81) n (%) | p value |
| --- | --- | --- | --- |
| **Demographics** |  |  |  |
| Male | 131 (52) | 43 (53) | 0.9 |
| Median age in years (range) | 68 (19-97) | 38 (0-85) |  |
| **Signs/Symptoms at admission** |  |  |  |
| Cough | 226 (90) | 74 (91) | 0.73 |
| Shortness of breath | 195 (78) | 59 (73) | 0.37 |
| Fever (T≥37.8°C) | 177 (71) | 63 (78) | 0.2 |
| Clinical suspicion of pneumonia | 180 (74) | 52 (64) | 0.09 |
| Sputum production | 137 (55) | 37 (46) | 0.2 |
| Chills | 126 (50) | 38 (47) | 0.6 |
| Feverish | 96 (38) | 25 (31) | 0.2 |
| Body ache | 75 (30) | 34 (42) | 0.04 |
| Wheezing | 64 (26) | 30 (37) | 0.05 |
| Nasal congestion | 60 (24) | 32 (40) | 0.01 |
| Nausea or vomiting | 65 (26) | 23 (28) | 0.7 |
| Headache | 59 (24) | 27 (33) | 0.08 |
| Sore throat | 51 (20) | 25 (31) | 0.05 |
| Diarrhea | 34 (14) | 13 (16) | 0.6 |
| **Outcomes** |  |  |  |
| Admission to intensive care unit | 101 (43) | 19 (30) | 0.05 |
| Intubation | 41 (22) | 12 (27) | 0.5 |
| Death | 25 (10) | 3 (3.7) | 0.07 |
| **Virology** |  |  |  |
| Influenza virus | 38 (15) | 11 (14) | 0.73 |
| Human metapneumovirus | 18 (7.2) | 7 (8.6) | 0.66 |
| >1 viral pathogen | 95 (34) | 39 (48) | 0.1 |

Supplemental Table 2. Comparison of patients hospitalized with severe acute respiratory infection with a viral pathogen to those with no viral pathogen identified, Arizona, 2010-2014

| Characteristics | SARI cases tested positive for viral infections   (n=134) n(%) | SARI cases tested negative for viral infections   (n=198) n(%) | p value |
| --- | --- | --- | --- |
| **Demographics** |  |  |  |
| Male | 64 (48) | 110 (56) | 0.16 |
| Median age in years (range) | 64 (0-97) | 63 (0-91) |  |
| **Signs/Symptoms at admission** |  |  |  |
| Cough | 125 (93) | 175 (88) | 0.14 |
| Shortness of breath | 103 (77) | 151 (76) | 0.90 |
| Fever (T≥37.8°C) | 104 (77) | 136 (69) | 0.07 |
| Clinical suspicion of pneumonia | 89 (67) | 143 (75) | 0.14 |
| Sputum production | 72 (54) | 102 (52) | 0.69 |
| Chills | 61 (46) | 103 (52) | 0.25 |
| Feverish | 48 (36) | 73 (37) | 0.85 |
| Body ache | 43 (32) | 66 (33) | 0.81 |
| Wheezing | 43 (32) | 51 (26) | 0.21 |
| Nasal congestion | 41 (31) | 51 (26) | 0.33 |
| Nausea or vomiting | 35 (26) | 53 (27) | 0.89 |
| Headache | 31 (23) | 55 (28) | 0.34 |
| Sore throat | 35 (26) | 41 (21) | 0.25 |
| Diarrhea | 18 (13) | 29 (15) | 0.76 |
| **Outcomes** |  |  |  |
| Admission to intensive care unit | 36 (32) | 84 (46) | 0.01 |
| Intubation | 18 (20) | 35 (24) | 0.49 |
| Death | 11 | 17 | 0.44 |
| **Influenza vaccination** | 56/98 (57) | 76/135 (56) | 0.90 |
